# Long Non-Coding RNAs: Key Regulators of Epithelial-Mesenchymal Transition, Tumour Drug Resistance and Cancer Stem Cells

**DOI:** 10.3390/cancers9040038

**Published:** 2017-04-21

**Authors:** Richard Heery, Stephen P. Finn, Sinead Cuffe, Steven G. Gray

**Affiliations:** 1 Thoracic Oncology Research Group, Rm 2.09, Trinity Translational Medical Institute, St. James’s Hospital, Dublin D08 W9RT, Ireland; rheery@tcd.ie; 2 Masters in Translational Oncology Program, Department of Surgery, Trinity College Dublin, Trinity Translational Medical Institute, St. James’s Hospital, Dublin D08 W9RT, Ireland; 3 Department of Histopathology & Morbid Anatomy, Trinity College Dublin, Dublin D08 RX0X, Ireland; Stephen.Finn@tcd.ie; 4 HOPE Directorate, St. James’s Hospital, Dublin D08 RT2X, Ireland; scuffe@stjames.ie; 5 Department of Clinical Medicine, School of Medicine, Trinity College Dublin, Dublin D02 R590, Ireland; 6 Labmed Directorate, St. James’s Hospital, Dublin D08 K0Y5, Ireland

**Keywords:** long non-coding RNA (lncRNA), epithelial-mesenchymal transition (EMT), chemotherapy resistance, cancer stem cell (CSC)

## Abstract

Epithelial mesenchymal transition (EMT), the adoption by epithelial cells of a mesenchymal-like phenotype, is a process co-opted by carcinoma cells in order to initiate invasion and metastasis. In addition, it is becoming clear that is instrumental to both the development of drug resistance by tumour cells and in the generation and maintenance of cancer stem cells. EMT is thus a pivotal process during tumour progression and poses a major barrier to the successful treatment of cancer. Non-coding RNAs (ncRNA) often utilize epigenetic programs to regulate both gene expression and chromatin structure. One type of ncRNA, called long non-coding RNAs (lncRNAs), has become increasingly recognized as being both highly dysregulated in cancer and to play a variety of different roles in tumourigenesis. Indeed, over the last few years, lncRNAs have rapidly emerged as key regulators of EMT in cancer. In this review, we discuss the lncRNAs that have been associated with the EMT process in cancer and the variety of molecular mechanisms and signalling pathways through which they regulate EMT, and finally discuss how these EMT-regulating lncRNAs impact on both anti-cancer drug resistance and the cancer stem cell phenotype.

## 1. Introduction

### Epithelial-Mesenchymal Transition: Cancer’s Gateway to Metastasis

Epithelial-mesenchymal transition (EMT) is a process whereby epithelial cells shed many of their epithelial traits and acquire various features of mesenchymal cells. During EMT, epithelial cells lose their polarity and many of their intercellular contacts such as desmosomes, adherens junctions and tight junctions, resulting in their disassociation from epithelial sheets. They subsequently assume a number of mesenchymal properties, including enhanced migratory capacity, invasiveness, heightened resistance to apoptosis and greatly increased production of extracellular matrix components [1,2,3,4].

The EMT program involves a multitude of molecular changes, including changes in the expression of cell-surface proteins, re-organization of the cytoskeleton and production of extracellular matrix (ECM)-degrading enzymes. In general, during EMT, expression of cell-adhesion molecule E-cadherin, desmosome protein desmoplakin, tight junction protein occluding, and intermediate filament protein cytokeratin are decreased, while expression of N-cadherin, intermediate filament protein vimentin and of several matrix metalloproteinases (MMPs) is increased [1,2,3,4]. A group of EMT-inducing transcription factors (EMT-TFs), notably Snail, Slug, ZEB1, ZEB2 and Twist, are activated during EMT and co-operate with each other to orchestrate these molecular changes [1,2,3,4]. The loss of expression of E-cadherin and the resulting detachment of epithelial cells from epithelial sheets is considered the major molecular event of EMT [1,2,3,4]. Snail, Slug, ZEB1, ZEB2 and Twist all bind to E-boxes within the E-cadherin promoter and repress the transcription of E-cadherin [5,6,7,8,9].

EMT occurs during both normal developmental, such as formation of the neural crest, and during normal physiological processes, such as wound healing [1,3]. However, carcinoma cells exploit the EMT program to become motile and invasive and EMT appears to be a critical process during the initiation of cancer metastasis [10,11,12]. E-cadherin is an important tumour suppressor and its expression is frequently lost in cancer via genetic mutation or epigenetic silencing of the *CDH1* gene encoding E-cadherin or alternatively via activation of various signalling pathways resulting in its downregulation [10,11,13]. In contrast to E-cadherin, N-cadherin expression promotes invasiveness and motility of cancer cells [13,14].

Signals from the tumour stroma, in particular TGF-β, EGF, FGF, PDGF and HGF, acting through downstream signalling pathways such as the TGF-β/SMAD, Wnt/β-catenin, MAPK/ERK, PI3K/Akt and Notch pathways, appear to be largely responsible for triggering EMT in carcinoma cells [2,12,15,16]. Acquired genetic mutations and epigenetic changes likely collaborate to make carcinoma cells far more responsive to EMT-inducing signals than normal epithelial cells [2,17].

The extent to which carcinoma cells pass through EMT varies, with some retaining many of their epithelial traits and others losing almost all traces of their former identity [2,10]. Carcinoma cells expressing markers of mesenchymal cells, such as vimentin, α-SMA, FSP1 and desmin, are frequently seen at the invasive fronts of tumours. These are believed to be tumour cells in the process of undergoing EMT and it is thought that these cells will subsequently enter into the invasion-metastasis cascade and ultimately give rise to metastatic disease [2,10].

## 2. Non-Coding RNA

### 2.1. The Non-Coding RNA Revolution

Following the sequencing of the human genome, the transcriptome could finally be analysed comprehensively. The major surprise of these efforts was that whilst only about 2% of the human genome codes for protein, the bulk of it is still transcribed into RNA, with estimates of the transcribed portion of the genome now ranging from 70% to 90% [18]. Thus, the vast majority of human RNA transcripts are non-coding. These non-coding RNAs ncRNAs are broadly divided into two categories according to their size: small ncRNAs less than 200 nucleotides long and long non-coding RNAs (lncRNAs) over 200 nucleotides long [19]. Small ncRNAs include well-characterized types like tRNAs and rRNAs as well as more recently discovered types such as miRNAs, siRNAs, snoRNAs, snRNAs and piRNAs which play a variety of cellular roles [20,21].

### 2.2. Long Non-Coding RNAs

LncRNA genes are broadly classified into five groups based on their location relative to the nearest protein-coding genes: (1) sense lncRNAs overlap one or more exons of a protein-coding gene on the coding strand of the gene; (2) antisense lncRNAs overlap exons of a protein-coding gene on the non-coding strand of the gene; (3) bidirectional lncRNAs are transcribed opposite the transcriptional start site of another transcript (4); intronic lncRNAs are completely contained within the introns of another transcript; and (5) long intergenic ncRNAs (lincRNAs) are located in between two protein-coding genes [22]. Over 100,000 lncRNAs have been identified to date in the human genome with the identification of new lncRNAs proceeding rapidly [23]. LincRNAs and sense lncRNAs are the two most abundant lncRNA types in humans, with lincRNAs accounting for nearly 60% and sense lncRNAs accounting for almost 25% of human lncRNAs in the LncRNAWiki database [23].

While once thought to merely represent transcriptional noise, the expression of lncRNAs has since been found to be cell type-specific and tightly regulated during development [24,25,26]. Although elucidating the function of lncRNAs has proved complex, as lncRNA function cannot presently be deduced from their sequence [27], it has become apparent that lncRNAs play highly diverse roles in the regulation of gene expression, being involved at the transcriptional, translational and post-translational levels of gene regulation [22,28,29,30].

**Figure 1:**
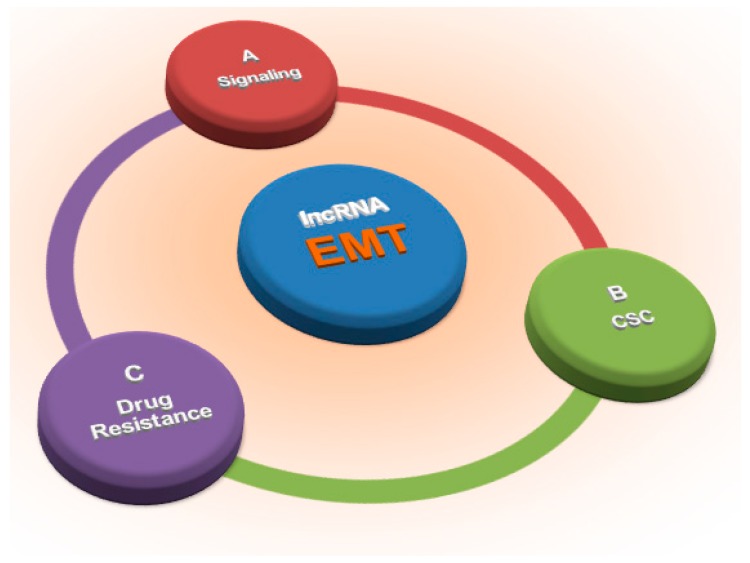
A generalized synopsis showing the links between lncRNAs and EMT with signalling pathways, cancer stem cells and drug resistance.

Aberrant expression of lncRNAs is now being realized to play a considerable role in tumourigenesis, with several hundred lncRNAs having been identified to be dysregulated in one or more human cancers so far [31,32,33]. Moreover, lncRNA dysregulation has already been found to contribute significantly to each of the six classic hallmarks of cancer cells: sustained proliferative signalling, evasion of growth suppressors, replicative immortality, invasion and metastasis, induction of angiogenesis and resistance to cell death [34]. In particular, an increasing number of lncRNAs are being implicated in the regulation of EMT acting to either promote (pro-EMT) or antagonize (anti-EMT) (Figure 1), often through acting as competing endogenous RNAs (ceRNAs) (Section 2.3) for miRNAs involved in EMT regulation (Section 2.4) or through mediating epigenetic silencing via the recruitment of the polycomb repressor complex 2 (PRC2) (Section 2.5).

### 2.3. Competing Endogenous RNAs

**Figure 2:**
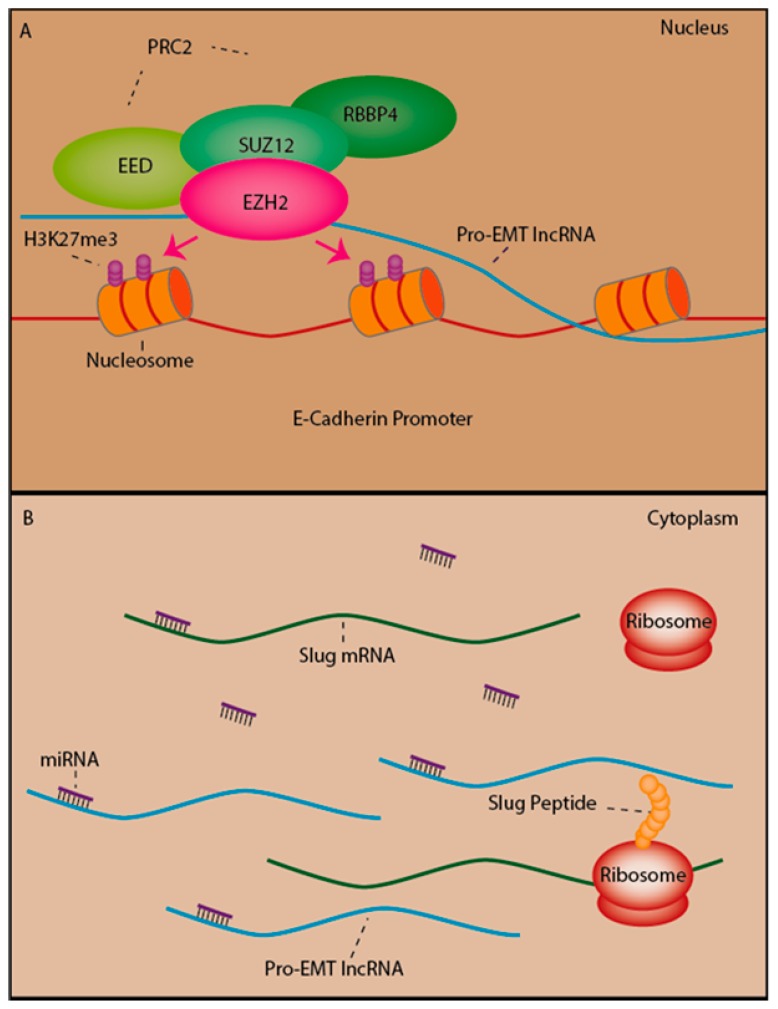
A depiction of two major gene regulatory mechanisms through which lncRNAs can promote epithelial mesenchymal transition (EMT), one taking place the nucleus and the other in the cytosol. (**A**) Recruitment of the PRC2 complex to the promoter of a gene which inhibits EMT (in this case E-cadherin) resulting in trimethylation of H3K27 (H3K27me3) and gene silencing; (**B**) Binding of pro-EMT lncRNAs to anti-EMT microRNAs, liberates translation of mRNA targets which induce EMT, (in this case Slug).

A major mechanism of gene regulation by ncRNAs that has recently been recognised is by acting as competing endogenous RNAs (ceRNAs) (Figure 2).

ceRNAs are RNA molecules (including both mRNAs and ncRNAs) that can bind and sequester or ‘sponge’ miRNAs by binding them via complementary miRNA response elements (MREs), thus liberating mRNA targets from negative regulation by these miRNAs [35,36,37]. Given that a substantial proportion of human mRNAs are predicted to be regulated by miRNAs [38,39], ceRNA activity could be responsible for indirect regulation of a significant fraction of human protein-coding genes. Indeed, competition between RNAs for miRNA binding may give rise to a highly complex gene regulatory system involving extensive crosstalk between the different species of RNA transcript [35]. Many lncRNAs possess MREs, with nearly 2000 miRNA-lncRNA interactions being experimentally verified in humans and millions more predicted, suggesting a wide-reaching role for lncRNAs in gene regulation through acting as ceRNAs [40]. Given the importance of certain miRNAs to the regulation of EMT (Section 2.4), it is unsurprising then that many lncRNAs are being found to govern EMT through acting as ceRNAs for these miRNAs (Section 3).

### 2.4. miRNAs and EMT

miRNAs are small ncRNAs approximately 21–23 nucleotides long that function in RNA silencing via complementary base-pairing to sequences within mRNA molecules [41,42]. It has become very apparent over the last decade that miRNAs are widely dysregulated in cancer and are instrumental in tumourigenesis [43,44,45]. Moreover, a set of miRNAs have been identified as being critical inhibitors of EMT. The miR-200 family, (comprising miR-200a, miR-200b, miR-200c, miR-141, and miR-429) is perhaps the best studied miRNA family with anti-EMT roles, with miR-200 members often highly expressed in epithelial cells compared to mesenchymal cells and downregulated during EMT [46,47]. All members of the miR-200 family target ZEB2, while miR-200a and miR-200b also target ZEB1 [46,47,48]. In turn, ZEB1 and ZEB2 inhibit transcription of miR-200 by binding to its promoter, creating a double negative feedback loop between ZEB1/ZEB2 and miR-200 [49]. In addition, miR-200b targets Slug with miR-200b and Slug also participating in a double negative feedback loop with each other [50].

miR-29 has been shown to negatively regulate EMT, possibly through targeting DNMT3A and DNMT3B and inhibiting de novo DNA methylation, including potentially the methylation of the E-cadherin promoter [51,52]. Numerous other anti-EMT miRNAs have been discovered: miR-1 and miR-204 target Slug [50,53], miR-29b, miR-30a and miR-34 all target Snail [54,55,56], miR-203 is involved in double negative feedback loops with both Snail and Slug [57,58], miR-205 targets ZEB1 and ZEB2 [48], miR-138 targets vimentin [59] and miR-194 targets N-cadherin [60]. The miRNA let-7, in addition to its other tumour suppressor roles, targets DNA-binding protein HMGA2 [61,62,63], which activates expression of Snail, Slug and Twist [64].A number of anti-EMT miRNAs are activated by p53 including miR-34a and miR-200 family members [65,66,67].

While the majority of the miRNAs regulating EMT identified thus far oppose EMT, several miRNAs promoting EMT have also been identified. miR-21, one of the most commonly upregulated miRNAs in solid cancers [68], is induced by TGF-β [69,70] and has been found to induce EMT in different cancer cell lines [70,71,72], with activation of Akt and ERK1/2 signalling seeming to be critical in the promotion of EMT by miR-21 [71,73]. Additionally, miR-9 targets E-cadherin and high levels of miR-9 are associated with cancer metastasis, while miR-155 promotes EMT by targeting RhoA GTPase, a key regulator of cell polarity and tight junction formation and stability [74,75].

### 2.5. LncRNAs as Mediators of Epigenetic Silencing Via PRC2 Recruitment

Polycomb repressive complex 2 (PRC2) is responsible for silencing genes through the trimethylation of lysine 27 on histone 3 (H3K27me3), a repressive histone modification which causes chromatin condensation and hinders transcription [76,77]. The core PRC2 complex is comprised of Embryonic Ectoderm Development (EED), the zinc finger protein Suppressor of Zeste, 12 (SUZ12), one of either of the histone binding proteins Retinoblastoma –Binding Protein 7 (RBBP7; also known as RbAp46) or Retinoblastoma-Binding Protein 4 (RBBP4; also known as RbAp48) and one of either of the histone methyltransferases Enhancer of Zeste homolog 1 or 2; (EZH1 or EZH2), which catalyse the trimethylation of H3K27 [76]. Thousands of lncRNAs have been identified to interact with PRC2, including roughly about 20% of studied lincRNAs [41,78] and genomic recruitment of PRC2 by lncRNAs has emerged as a major mechanism of gene regulation by lncRNAs [78,79]. The best understood example of lncRNA-mediated recruitment of PRC2 is in the process of X chromosome inactivation by the lncRNA Xist. Xist coats the X chromosome from which it is transcribed and recruits PRC2 to this chromosome, leading to lasting epigenetic silencing of one X chromosome [80,81]. In a similar manner, oncogenic lncRNAs are now being found to contribute to tumourigenesis and metastasis by their actions in silencing tumour suppressor genes via the recruitment of PRC2 [82,83,84].

PRC2, and in particular its associated subunit EZH2, have emerged as key regulators of EMT (Figure 2). Epithelial cells generally express very low levels of EZH2, while increased levels are often observed in cancer where EZH2 promotes EMT, invasiveness and metastasis via silencing of the E-cadherin promoter by H3K27 trimethylation, with knockdown of EZH2 able to reverse both the H3K27 trimethylation of the E-cadherin promoter and EMT [85,86,87]. Snail1 was shown to interact with Suz12 and EZH2 to recruit PRC2 to the E-cadherin promoter and the silencing of E-cadherin by Snail1 was in fact found to depend on PRC2 activity, indicating the regulation of E-cadherin by PRC2 is a critical process during activation of EMT [88]. Several lncRNAs have now been found to regulate EMT by recruiting PRC2 or components of this complex to the E-cadherin promoter and/or the promoters of other genes involved in regulating EMT (discussed in more detail in Section 3 and summarized in Table 1).

## 3. LncRNAs and EMT: The Main Players

Most of the lncRNAs regulating EMT identified thus far can broadly be divided into those which promote EMT (pro-EMT) lncRNAs and those which suppress EMT (anti-EMT) lncRNAs, with most falling into the pro-EMT category (Table 1). However, the role of some lncRNAs in EMT is more complicated, as in some cancers individual lncRNAs may function as pro-EMT whilst in other cancers the same lncRNAs may possess anti-EMT activity.

### 3.1. Pro-EMT

#### 3.1.1. LncRNAs with Pro-EMT Activity

MALAT-1 (metastasis associated lung adenocarcinoma transcript 1; also, called NEAT2 or nuclear enriched abundant transcript 2) was one of the first lncRNAs to be associated with tumourigenesis and was identified as a predictive marker for the development of metastatic disease and shorter survival in early stage lung adenocarcinoma—lung adenocarcinomas expressing high levels of MALAT-1 display almost 5 times the risk of metastasis as tumours with low expression [274]. MALAT-1 is overexpressed and has been linked to the promotion of EMT in bladder cancer, cervical cancer, NSCLC, pancreatic cancer and renal cell carcinoma [91,274,275,276,277,278,279]. However, contradictory results have been obtained for the effect of MALAT-1 on EMT in breast cancer cells, with two studies reporting the promotion of EMT by MALAT-1 [89,280] and another reporting inhibition of EMT by MALAT-1 [97].

MALAT-1 has been shown to regulate EMT in various ways. By acting as ceRNA for miR-1 and miRNA-204, this results in the de-repression of Slug, a common target of both miRNAs, and promotes EMT [90,281,282] (Figure 2). MALAT-1 has also been shown to act as a ceRNA for miR-205 [91], a miRNA known to target both Zeb1 and Zeb2 [48]. Another mechanism by which MALAT-1 induces EMT is by recruiting PRC2 components Suz12 and EZH2 to the E-Cadherin promoter [91,93].

H19 is an imprinted oncofetal lncRNA, and has long been identified as an aberrantly expressed non-coding RNA in a great number of cancers with multi-faceted roles throughout tumourigenesis [283]. H19 is upregulated by a number of signals inducing EMT, including TGF-β, hypoxia and HGF, suggesting that it may therefore play a pivotal role in the activation of EMT [284]. H19 is also activated by Slug with Slug and H19 forming a positive feedback loop [284]. Overexpression of this lncRNA is associated with the activation of EMT in numerous cancers, including pancreatic cancer, CRC, nasopharyngeal carcinoma, bladder cancer, gallbladder cancer and oesophageal cancer4 [101,102,104,105,285,286]. H19 has been found to silence E-cadherin through recruitment of EZH2 to its promoter [105]. H19 also functions as a ceRNA for several pro-EMT miRNAs, including let-7, miR-138, miR-200 family members miR-141 and miR-200a and miR-630, and by doing so, promotes EMT through derepression of the let-7 target HMGA2 [101,102], the miR-138 target vimentin [102], the miR-200 targets Zeb1 and Zeb2 [102,103], and the miR-630 target EZH2 [104].

H19 has itself also been shown to contain a miRNA (miR-675) [287], and when processed this miRNA has also been shown to have functional roles in EMT. For instance, miR-675 is necessary for the upregulation of Slug and downregulation of E-cadherin by H19 in breast cancer cells [284]. Furthermore, in a model designed to recapitulate EMT/MET switching during colon cancer metastasis, elevated levels of H19 have been observed in cells isolated from metastases, and indeed levels of miR-675 were also increased in these cells [288].

Loss or decreased expression of miR-675 has been shown to occur in melasma, and is associated with the regulation of its target cadherin 11 (CDH11). This loss of miR-675 results in the overexpression of CDH11 resulting in the induction of N-cadherin and Twist1 expression, and a concomitant decrease of E-cadherin expression [289]. Moreover, in hepatocellular carcinoma (HCC), miR-675 has been shown to directly promote E-cadherin expression through directly targeting of Twist1 in HCC cells [290].

LncRNA-ATB (lncRNA Activated by TGF-β), has recently been shown to be overexpressed and to promote EMT in colon cancer, gastric cancer, lung cancer HCC, prostate cancer and RCC [169,170,172,173,291,292]. One of the identified functions of LncRNA-ATB is its ability to act as a ceRNA for the miR-200 family, leading to derepression of miR-200 EMT-associated targets genes ZEB1, ZEB2, ZNF217 and TGF-β2 [167,168,169]. More recently, in gastric cancer, this lncRNA has also been shown to function as a ceRNA competing with TGF-β2 for binding of miR-141-3p [171].

However, in contrast to the above findings in other malignancies, lncRNA-ATB was found to be downregulated in pancreatic cancer, with low levels being associated with advanced and metastatic disease [293]. Likewise, miR-200a and miR-200b, have been shown to be overexpressed in pancreatic cancer despite being downregulated in many other cancers [294]. As such, it would appear that an lncRNA-ATB-miR-200-ZEB1/2 axis appears to play a critical tumour suppressor role in pancreatic cancer compared to the tumourigenic role it plays in most other cancers.

The lncRNA Highly Upregulated in Liver Cancer (HULC) is one of the most upregulated genes in HCC [295] and was also shown to be upregulated by the Hepatitis B virus X (HBx) protein, which plays a prominent role in the pathogenesis of HBV-associated HCC [296]. HULC overexpression has been found to upregulate Snail in HepG2 HCC cells [297]. HULC has also been reported to induce EMT in gastric cancer [298]. HULC activates EMT in HCC part by acting as a ceRNA for miR-200a and upregulating Zeb1 [175]. Additionally, HULC acts as a ceRNA for miR-372 [177]. In chronic myeloid leukemia (CML), whilst not associated with EMT, HULC was also shown to act as a ceRNA for miR-200a [176]. While mir200a has been shown to target TGFBR2 and the RhoGTPase RhoC and to block TGF-β-induced EMT [299], the study by Lu et al., suggest that HULC can also act to inhibit c-Myc expression and PI3K/Akt signalling [176].

UCA1 induces EMT in bladder cancer cells by upregulating the expression of ZEB1 and ZEB2. It achieves this by acting as a ceRNA for miR-145, which (a) directly inhibits ZEB2; and (b) indirectly inhibits ZEB1 via targeting Oct4, a transcriptional activator of ZEB1 [185]. UCA1 has also been shown to suppress metastasis of epithelial ovarian cancer by functioning as a ceRNA for miR-485-5p [188], a miRNA which blocks EMT and metastasis through its targeting of MMP14 and HMGA2 [188,300]. UCA1 has also been shown to regulate EMT in Gastric Cancer as, silencing of UCA1 resulted in decreased levels of vimentin and snail, with concomitant elevated levels of E-cadherin [196]. It has also been shown to induce EMT in breast cancer by enhancing Wnt/beta-catenin signalling pathway, and again silencing of UCA1 resulted in increased expression of E-cadherin but decreased expression of N-cadherin, Vimentin and Snail [192]. UCA1 has been shown to play important roles in drug resistance in some instances by directly regulating miRNAs or by functioning as a ceRNA for various miRNAs [184,186,197]. It can however, also act to induce chemo-resistance via its activities on EMT [189,190,194].

Like UCA1, another lncRNA Taurine upregulated gene 1 (TUG1), also promotes EMT and invasion in bladder cancer through acting as a ceRNA for miR-145 and liberation of expression of the miR-145 target ZEB2 [204]. Additionally, knockdown of TUG1 was able to reduce lung metastasis of bladder cancer cells in vivo [204]. TUG1 has most recently been shown to act as a ceRNA for miR-300 to regulate EMT in gallbladder cancer [205]. TUG1 also appears to contribute to invasion and metastasis in colorectal cancer and cervical cancer through promotion of EMT [301,302,303].

NEAT1 (nuclear enriched abundant transcript 1) has been shown to promote EMT in nasopharyngeal carcinoma, and gastric cancer [208,304]. NEAT1 knockdown in ovarian cancer cells resulted in a decrease in Snail1, TGF-β1, MMP-2 and MMP-9 and so presumably promotes EMT in ovarian cancer [305]. NEAT1 has been found to act as a ceRNA for at least two miRNAs. NEAT1 binding to miR-204 results in depression of the miR-204 target Zeb1 and promotion of EMT [208]. Alternatively, binding of miR-101 by NEAT1 leads to derepression of miR-101 target EZH2 [209], and given EZH2’s prominent role in EMT induction, this very likely contributes to induction of EMT by NEAT1.

A role for the LncRNA-EBIC (EZH2-binding lncRNA in cervical cancer) and EMT has been described for cervical cancer. This lncRNA has been shown to associate with EZH2 and appears to facilitate the silencing of E-cadherin by EZH2 in cervical cancer. Knockdown of lncRNA-EBIC impaired migration and invasion of cervical cancer cells in vitro [215].

The lncRNA Colon cancer-associated transcript-1 (CCAT1), also known as Cancer-associated region long non-coding RNA (CARLo-5) was originally identified in colon cancer [306], but has since been shown to be overexpressed in several other cancers [216,217,218,220,221,306,307,308,309,310,311]. This lncRNA has been shown to be associated with the c-MYC enhancer to drive the overexpression of this lncRNA in colorectal cancer [220]. In NSCLC, however, this lncRNA has been shown to be associated with the regulation of EMT as abrogation with siRNA resulted in the reversion of EMT in an NSCLC cell line, which increased expression of E-cadherin while downregulating expression of vimentin, fibronectin Snail and Twist [307].

Colon cancer associated transcript 2 (CCAT2) appears to promote EMT, as lncRNA CCAT2 knockdown leads to increased E-cadherin and decreased vimentin levels in gastric cancer cells and, additionally, CRC cells expressing higher levels of CCAT2 display increased vimentin expression [312,313]. The expression of this lncRNA has now been associated with metastasis in many cancers [224,226,314,315,316,317,318,319,320,321,322,323,324]. The precise mechanism by which CCAT2 regulates EMT are currently unknown, but in gastric cancer CCAT2 interacts with EZH2 and promotes EMT by downregulation of E-cadherin expression and upregulation of ZEB2, LATS2, Vimentin and N-cadherin [224], and similar effects of CCAT2 expression on E-cadherin, N-cadherin and vimentin were observed in prostate cancer [323].

Plasmacytoma variant translocation 1 (PVT1) is a lncRNA transcribed from the 8q24 chromsomal region which also harbors the c-Myc oncogene and is among the most frequently amplified chromosomal sites in human cancers, with almost 20% of tumours being found to exhibit increased copy number for both *c-Myc* and PVT1 [325]. PVT1 induces EMT and migration in oesophageal and pancreatic cancer cells with increased expression associated with advanced disease stage and metastasis [326,327]. In hepatic stellate cells, PVT1 was shown to promote EMT and activate Hedgehog signalling by acting as a ceRNA for miR-152 and promoting methylation of the promoter of PTCH1, a negative regulator of Hegehog signalling [328].

#### 3.1.2. Antisense lncRNAs

A number of antisense lncRNAs have also been found to promote EMT (Table 1). Both the ZEB1 and ZEB2 genes are associated with antisense lncRNA genes, ZEB1-AS1 and ZEB2-AS1 respectively [154,329]. ZEB1-AS1 is upregulated and associated with shorter overall survival and higher recurrence rates in both HCC and oesophageal squamous cell carcinoma [329,330]. Overexpression of ZEB1-AS1 in HCC cells resulted in upregulation of ZEB1, induction of EMT, increased invasiveness and proliferation in vitro and increased tumour growth and metastasis in vivo [329]. Overexpression of ZEB1-AS1 has also been found in gliomas [331], and osteosarcomas [152,153]. In gliomas, ZEB1-AS1 activates EMT by up-regulating the expression of ZEB1, MMP2, MMP9, N-cadherin, and Integrin-beta1 as well as decreasing E-cadherin levels [331].

ZEB2-AS1 (also called ZEB2 NAT) is induced by Snail1 and promotes translation of ZEB2 mRNA. ZEB2-AS1 is complementary to a 5′ splice site of an intron in the 5′ UTR of the ZEB2 mRNA containing an internal ribosome entry site (IRES) that is necessary for efficient translation of the ZEB2 mRNA. Binding of ZEB2-AS1 prevents the splicing of this intron, resulting in increased translation and upregulation of the Zeb2 protein [154]. ZEB2-AS1 has been found to be upregulated in urinary bladder cancer [155] and HCC [332], and to be partly responsible for the activation of ZEB2 during EMT induction by TGF-β1 in urinary bladder cancer cells [155]. Knockdown of ZEB2-AS1 in HCC cells results in reduced vimentin and N-caherin expression with restoration of E-cadherin expression [332].

EGFR-AS1 is another antisense lncRNA and as its name suggests it is transcribed from the EGFR locus. This lncRNAis upregulated in HCC [156] and its upregulation is associated with portal vein thrombosis, lymph node metastasis and poorer overall survival following surgery. Knockdown of EGFR-AS1 led to decreased expression of EGFR and suppression of migration and EMT in HCC cells [156].

Another similar lncRNA, HNF1A-AS1, is transcribed antisense to the HNF1A gene and is overexpressed in lung adenocarcinoma and oesophageal adenocarcinoma [159,160]. Knockdown of HNF1A-AS1 led to increased E-cadherin expression and decreased N-cadherin and β-catenin levels in lung adenocarcinoma cells [159]. HNF1A-AS1 was shown to interact with DNMT1, and as DNMT1 is known to silence E-cadherin during tumourigenesis [157,158], it is very possible that HNF1A-AS1 silences E-cadherin via the recruiting DNMT1 to the E-cadherin promoter, though this remains to be investigated. Interestingly, HNF1A-AS1 knockdown was found to lead to reduction in H19 expression, while H19 knockdown did not affect HNF1A-AS1 expression, indicating HNF1A-AS1 is an upstream regulator of H19 [160].

CBR3-AS1 (also called prostate cancer-up-regulated long noncoding RNA 1 or PlncRNA1), is an lncRNA transcribed antisense to the carbonyl reductase 3 gene. CBR3-AS1 is overexpressed in HCC and knockdown of CBR3-AS1 was shown to reduce invasion and migration in the HCC cell line HCCLM3 by preventing EMT [333]. Its expression has also been shown to be elevated in gastric cancer [334], esophageal squamous cell carcinoma (ESCC) [335], and prostate cancer [164]. Indeed, expression of CBR3-AS1 and the AR are tightly linked as CBR3-AS1 was initially shown to directly regulate AR expression [336], and more recently this lncRNA was shown to regulate the AR by protecting it from microRNA (miR-34c and miR-297) mediated suppression in prostate cancer cells [165]. CBR3-AS1 is tightly correlated with TGF-β levels in HCC and together they promote EMT [164].ZFAS1 (zinc finger antisense 1), also called ZNFX1-AS1, is an lncRNA transcribed antisense to the *ZNFX1* protein-coding gene. It was first identified as an lncRNA involved in mammary development and was found to be downregulated in breast cancer [337]. However, ZFAS1 has been shown to be pro-tumourigenic and promote EMT in a number of other cancers, including colon cancer, gastric carcinoma and glioma [234,338,339].

How antisense lncRNAs such as ZEB1-AS1 and EGFR-AS1 induce expression of the sense protein-coding genes is not currently yet known. However, antisense transcription appears to be capable of enhancing transcription of downstream sense genes via several distinct mechanisms, including the displacement of nucleosomes, localization of RNA polymerase in the vicinity of the sense promoter, promotion of negative supercoiling of DNA and prevention of the spread of repressive chromatin [340]. Another possibility is that, like ZEB2-AS1, the antisense lncRNAs could regulate translation of the sense gene’s mRNA. Finally, they could potentially involve mechanisms similar to the way lncRNAs have been found to regulate genomic imprinting [341], but this remains to be resolved.

#### 3.1.3. LincRNAs

Several lincRNAs have been found to promote EMT. HOTAIR is a lincRNA transcribed from the HOXC gene cluster and promotes epigenetic silencing of target genes, including the HOXD gene cluster, through the recruitment of PRC2, which interacts with HOTAIR’s 5′ end, and of the LSD1/CoREST/REST lysine demethylase complex, which interacts with HOTAIR’s 3′ end [342,343]. HOTAIR is overexpressed in a wide variety of solid malignancies and high expression of HOTAIR is strongly associated with metastasis and tumour recurrence in many of these [344,345]. The HOTAIR lincRNA has been extensively linked to promotion of EMT, and has been shown to be involved with EMT in bladder cancer, breast cancer, cervical cancer, colon cancer, gastric cancer, epithelial ovarian cancer and oral squamous cell carcinoma [115,116,346,347,348,349,350].

Like H19 and MALAT-1, HOTAIR has been found to silence E-cadherin through recruitment of PRC2 to the CDH1 promoter [115]. HOTAIR has recently been shown to form a tripartite complex with Snail and EZH2, facilitating the recruitment of EZH2 to Snail binding sites in the promoters of epithelial genes E-cadherin, HNF1α and HNF4α, leading to their epigenetic silencing [120]. HOTAIR also mediates the epigenetic silencing of the anti-EMT miRNAs miR-34a and miR-568 [115,116]. In this regard, HOTAIR recruits PRC2 to the miR-34 promoter, leading to miR-34 silencing and upregulation of the miR-34a targets Snail and the HGF receptor c-Met, promoting EMT [116]. HOTAIR also silences miR-568 via recruitment of both EZH2 and LSD1 to the miR-568 promoter in breast cancer cells, leading to derepression of the miR-568 target NFAT5, which induces of EMT and invasion in breast cancer via transcriptional activation of calcium binding protein S100A4 [117]. Additionally, HOTAIR indirectly regulates expression of the anti-EMT miRNA miR-7 through its epigenetic silencing of HoxD10 [119]. miR-7 targets the SETDB1 histone methyltransferase, leading to downregulation of STAT3 and subsequently decreased activation by the STAT3 pathway of c-Myc, Twist and miR-9 (which represses E-cadherin mRNA). HoxD10 is involved in the transcriptional activation of pre-miR-7-1, and as such silencing of HoxD10 by HOTAIR leads to decreased levels of miR-7 [119]. HOTAIR has recently been shown to form a tripartite complex with SNAIL and EZH2 to SNAIL regulated EMT genes [120].

Linc-regulator of reprogramming (ROR) is another lincRNA known to regulate EMT. linc-ROR is upregulated and induces EMT in both breast [133] and pancreatic cancer [135]. Overexpression of linc-ROR promotes metastasis of breast cancer cells in vivo while linc-ROR knockdown hindered metastasis [133]. linc-ROR activates EMT in part by acting as a ceRNA for the anti-EMT miRNAs miR-205, which results in derepression of ZEB1 and ZEB2 [133], and acts as a ceRNA for miR-145 [132]. This miRNA normally targets ZEB2, and this double negative feedback loop regulates epithelial-mesenchymal transition in prostate cancer cells [351].

A lincRNA located nearby to the TCF7 gene, called lncTCF7, is highly expressed in HCC tumours [140], and in NSCLC [142]. Described initially as an lncRNA, it is intergenic and as such is a lincRNA and plays important roles in EMT induction in both HCC [141] and NSCLC cells [142]. lncTCF7 is strongly induced in HCC cells by IL-6/STAT3 signalling and appears to be highly important for the induction of EMT by IL-6 [141].

Lastly, linc00152 is upregulated in various cancers including gastric cancer [145,147,352,353], lung cancer [144], gall bladder cancer [148,149], renal cell carcinoma [354], and pancreatic cancer [355], whilst its expression has been found to be downregulated in colorectal cancer [356]. linc00152 can act via a ceRNA network for miR-139-5p in gastric cancer [143], and acts as a competing endogenous RNA to modulate the expression of miR-193a-3p in colon cancer [151], and acts as a ceRNA for miR-138 in gallbladder cancer [148]. Knockdown of linc00152 was found to reverse EMT in gastric cancer cells [353].

### 3.2. Anti-EMT LncRNAs

#### 3.2.1. lncRNAs

Expression of the lncRNA growth arrest-specific transcript 5 (GAS5) is reduced in several cancers [347,357,358,359], and re-expression of this lncRNA is associated with anti-proliferative activity. One of the mechanisms by which GAS5 functions as a tumour suppressor in HCC is via its ability to impede EMT. Overexpression of GAS5 in HCC cells resulted in decreased vimentin levels and increased E-cadherin levels and in significant repression of the invasion and proliferation of HCC cells in vitro [360]. Additionally, an lncRNA transcribed anti-sense to GAS5, GAS5-AS1, has also been found to be downregulated in NSCLC and inhibit EMT, migration and invasion of NSCLC cells [361]. Thus, the GAS5/GAS5-AS1 axis appears to be highly important to the regulation of EMT.

LncRNA-Down-Regulated Expression by HBx (Dreh) is an lncRNA downregulated by the HBV protein HBx and is frequently downregulated in HBV-related HCC [243], with low expression of lncRNA-Dreh being associated with poor prognosis. LncRNA-Dreh acts as a tumour suppressor in HBV-induced HCC, inhibiting HCC cell growth in vitro and metastasis of HCC cells in vivo. LncRNA-Dreh was found to interact with vimentin protein and alter the structure of vimentin intermediate filaments, as well as to repress vimentin protein expression and thus likely functions as a tumour suppressor at least in part through repression of EMT [243]. Therefore, HBx regulates two lncRNAs governing EMT, downregulating anti-EMT lncRNA-Dreh and upregulating pro-EMT lncRNA HULC [296]. It should be noted that HBx also regulates other important pro-tumourigenic lncRNAs such as DBH-AS1 [362,363,364].

Tumour suppressor candidate 7 (TUSC7) is downregulated in colorectal cancer, glioma, HCC, NSCLC and osteosarcoma [244,245,246,365,366]. In HCC TUSC7 has been demonstrated to prevent EMT, invasion and metastasis by serving as a ceRNA for miR-10a, which is upregulated in HCC and associated with EMT promotion. Inhibition of miR-10a by TUSC7 led to increased expression of the miR-10a target EphA4, which has been identified to suppress EMT in cancer through regulating integrin β1 pathway signalling [244]. TUSC7 has also been shown to act as a ceRNA for miR-211-3p [245], to specifically bind to miR-23b [246] and TUSC7 has been shown to directly repress miR-23b expression in GC [247], but whether these effects play roles in the regulation of EMT have yet to be determined.

BRAF activated non-coding RNA (BANCR) is an lncRNA frequently downregulated in many cancers [270,272,367,368,369]. When downregulated it has been shown that BANCR can act in a capacity as an anti-EMT. In this regard, expression of BANCR is reduced in patient tumours, and re-expression of BANCR levels induced E-cadherin expression, along with decreased N-cadherin, Vimentin, SNAIL1, and SNAIL2 expression [270,369]. It must be noted however, that overexpression of BANCR has also been found to occur in cancer [367,370,371,372].

LncRNA amine oxidase, copper containing 4, pseudogene (AOC4P), has been found to be downregulated in HCC and inhibits EMT in HCC cells by promoting vimentin’s ubiquitination and degradation, and overexpression of AOC4P is associated with decreased cell migration in vitro and reduced metastasis in vivo [250].

Another lncRNA with an anti-EMT role is LEIGC. This lncRNA was found to be down-regulated in gastric cancer. LEIGC overexpression could significantly impair the motility of gastric cancer cells in vitro and LEIGC knockdown promoted tumour progression in vivo [251]. Knockdown of LEIGC by shRNA resulted in downregulation of epithelial cell-related genes such as CDH1, whereas several mesenchymal cell markers (such as snail, slug, zeb, and twist) were upregulated, while overexpression of LEIGC demonstrated the opposite effect [251]. For many of these anti-EMT lncRNAs, the mechanisms by which they inhibit EMT are currently unknown.

#### 3.2.2. lincRNAs

LincRNA-p21 is an lncRNA located about 15 kb upstream of the p21/Cdkn1a gene and is directly induced by p53 [373]. Its expression has been shown to be downregulated in prostate cancer [258], NSCLC [374] and HCC [255]. LincRNA-p21 has been shown in HCC to inhibit EMT via Notch signalling [255], but it plays many other additional regulatory roles [254,257,373,375].

Other lincRNAs associated with an anti-EMT activity include SLC25A25-AS1 which is downregulated in both the tumours and serum of CRC patients [252], and CPS1-IT1 (carbamoyl-phosphate synthase 1 intronic transcript 1) downregulated in the majority of HCC and NSCLC tumours [262,376]. Finally, linc00261 another lincRNA with an anti-EMT activity is downregulated in choriocarcinoma [377], gastric cancer [353,378,379] and pancreatic cancer [355] and in part this lincRNA suppresses EMT by causing the degradation of Slug [263].

### 3.3. LncRNAs with Varying Roles in EMT Regulation in Different Cancers

Several lncRNAs have demonstrated contrasting roles in the regulation of EMT that are dependent on the cancer of origin. A classic example is that of BANCR which has been found to be overexpressed and to induce EMT and migration in CRC and HCC [270,372], while being downregulated and inhibiting EMT in NSCLC [369].

Other examples of this complexity include SPRY4-IT1, which is derived from an intron of SPRY4, is upregulated in many cancers and has been shown to induce EMT in colorectal cancer, gliomas, HCC, oesophageal squamous cell carcinoma and osteosarcoma [264,266,267,380,381,382,383,384]. In bladder cancer, SPRY4-IT1 is upregulated [385] and induces metastasis whereby it acts as a ceRNA for miR-101-3p, resulting in up-regulation of EZH2 [265].

In contrast, SPRY4-IT1 is downregulated in NSCLC and prevents EMT in NSCLC cells [386]. SPRY4-IT1 downregulation in NSCLC appears to be brought about by EZH2. In EZH2-knockdown NSCLC cells, which display reduced metastatic potential and decreased levels of EMT markers, the inhibition of SPRY4-IT1 could partially restore invasiveness and reverse the downregulation of EMT markers. Therefore, SPRY4-IT1 silencing appears to play a significant role in EZH2-mediated induction of EMT and tumourigenesis in NSCLC [386]. Interestingly, SPRY4 itself has also been shown to repress EMT in NSCLC and thus the SPRY4 locus, including both SPRY4 and SPRY4-IT1, appears to be highly important to the regulation of EMT in lung cancer cells [387].

Another lincRNA that shows similar tissue context differences in roles is Linc01133, which is upregulated in NSCLC [388] and promotes EMT [269], while in CRC its expression is reduced and it functions to inhibit EMT in CRC cells [268].

One potential confounding issue with these sorts of observations has been the potential issue of reproducibility. For instance, different studies in gastric cancer have produced contradictory results, with one study reporting upregulation of SPRY4-IT1 [389] and another reporting downregulation of SPRY4-IT1 [390].

## 4. LncRNAs in Signalling Pathways Governing EMT

Regulation of EMT involves a complex signalling network involving various pathways, the principal pathways currently identified being TGF-β, Wnt/β-catenin, MAPK/ERK, PI3K/Akt, hypoxia/HIF and Notch [391,392] (Table 1). In the following sections, we discuss how lncRNAs associated with EMT are being found to both regulate signalling via these pathways as well as functioning as downstream effectors of them.

### 4.1. TGF-β Signalling

The TGF-β signalling pathway is one of the major pathways responsible for activation of EMT during tumourigenesis, inducing expression of Snail, Slug and Twist via activation of the Smad family of transcription factors and HMGA2 [64,393,394]. The expression of hundreds of lncRNAs is regulated during activation of EMT by TGF-β [230,395]. Many of the lncRNAs upregulated by TGF-β seem to be important in the downstream activation genes involved in EMT, including expression of EMT associated TFs (Table 1). LncRNA-ATB is one of the most upregulated lncRNAs in HCC cells following TGF-β treatment and is also upregulated in other cancers such as breast, gastric cancer, prostate and renal cell carcinoma [168,169,170,172,173,292]. Knockdown of this lncRNA has been shown to impede the induction of EMT by TGF-β in various cells [169,172]. Through acting as a ceRNA for miR-200 family members, lncRNA-ATB promotes expression of TGF-β2 and zinc finger protein ZNF217, a transcriptional activator of TGF-β [167,168,169,173,396]. Thus, lncRNA-ATB and TGF-β appear to form a positive feedback loop, with TGF-β inducing lncRNA-ATB and lncRNA-ATB amplifying TGF-β signalling.

LncRNA-HOXA transcript induced by TGF-β (HIT) is one of the most upregulated lncRNAs in mouse mammary epithelial cells following TGF-β treatment [230]. Knockdown of lncRNA-HIT attenuates EMT activation in mouse mammary epithelial cells in response to TGF-β, suggesting it is highly important to the activation of EMT by TGF-β signalling. Furthermore, lncRNA-HIT knockdown decreased the migratory and invasive capacity of mouse mammary tumour cells and inhibited lung metastasis in a mouse orthotopic breast cancer model. Expression of lncRNA-HIT was found to increase during breast cancer progression, being low in normal human breast tissue, increased in breast hyperplasia and ductal carcinoma in situ and highest in invasive ductal carcinoma [230]. Most recently lncRNA-HIT has been shown to be upregulated in NSCLC and to play a role in inducing cell migration, invasion, tumour growth, and metastasis through stabilization of ZEB1 protein [229].

Several other major pro-EMT lncRNAs are also induced by TGF-β signalling and seem to be important to induction of EMT by TGF-β. For instance, ZEB2-AS1 is upregulated in bladder cancer cells in response to TGF-β1 secreted by cancer-associated fibroblasts and partially mediates EMT and the acquisition of invasiveness in bladder cancer cells exposed to TGF-β1 [155]. As ZEB2-AS1 itself regulates the translation of ZEB2 mRNA [154], ZEB2-AS1 likely links TGF-β1 signalling and increased ZEB2 expression. Another lncRNA, MALAT-1, is induced by TGF-β and plays a critical role during the promotion of EMT by TGF-β in bladder cancer cells [93]. In a similar vein, HOTAIR was found to be essential to the induction of EMT by TGF-β1 in colon cancer and breast cancer cells [121]. H19 is also an lncRNA found to be upregulated by TGF-β [106,107,397] and TGF-β mediated upregulation of Slug is dependent in part by this lncRNA [106,284]. Conversely, linc01133, which inhibits EMT in CRC, has been found to be downregulated in response to TGF-β signalling in CRC [268]. It is clear that lncRNAs play critical roles in both TGF-β signalling and its regulation of EMT and the numbers of lncRNAs associated with this continue to grow with recent additional examples include UCA1 [196], MEG3 [398] and BX357664 [399].

### 4.2. Wnt/β-Catenin Signalling

The Wnt/β-Catenin signalling pathway is also critical to activation of EMT in cancer [400,401], and often its pathways converge with TGF-β signalling to regulate EMT [402]. As a significant subset of the cellular pool of β-catenin molecules in a cell are bound to the cytoplasmic domain of E-cadherin, the subsequent downregulation of E-cadherin during EMT therefore results in the liberation of these β-catenin molecules and their freedom to travel to the nucleus and activate EMT-associated genes as part of the canonical Wnt signalling pathway [401,403]. Several lncRNAs implicated in EMT regulation also affect Wnt/β-Catenin signalling. For example, HOTAIR epigenetically silences the Wnt inhibitor WIF1, leading to increased Wnt signalling in oesophageal squamous cell carcinoma [118]. MALAT-1 was also demonstrated to induce EMT in squamous cell carcinoma of the tongue via the Wnt/β-catenin signalling pathway [94], while MALAT-1 knockdown in renal cell carcinoma decreased expression of β-catenin and transcription factor c-Myc, a downstream effector of Wnt/β-Catenin signalling [94]. Furthermore, loss of WIF1 was shown to enhance the migratory potential of glioblastoma cells through WNT5A activation mediated via MALAT1 [404].

H19 has been found to epigenetically silence the promoter of the Wnt pathway antagonist NKD1 through EZH2 recruitment, leading to increased Wnt signalling [105]. lncTCF7 recruits the Swi/SNF nucleosome remodelling complex to the TCF7 promoter, triggering TCF7 expression and leading to activation of Wnt pathway signalling [140]. The lncRNA UCA1 plays critical roles in the regulation of EMT via the Wnt signalling pathway [189,190,191,192,193]. In contrast, lincRNA-p21 negatively regulates expression and activity of β-catenin [253,254]. As such, Wnt signalling and the regulation of this pathway via lncRNAs is important for EMT (Table 1).

### 4.3. MAPK/ERK Signalling

In addition to their roles in EMY via TGF-β signalling several lncRNAs also utilize the MAPK/ERK signalling pathway in EMT (Table 1). For instance, the lncRNA UCA1 also acts through MAPK/ERK signalling. In this regard, UCA1 contributes to HCC tumourigenesis in part through acting as a ceRNA for miR-216b, leading to depression of fibroblast growth factor receptor 1 (FGFR1) and consequent activation of MAPK/ERK signalling [187]. As activation of FGFR1 has been documented to promote EMT in other cancers [405,406,407], it is likely that induction of FGRF1 signalling by UCA1 could promote EMT. Likewise, it has been suggested that lncRNA SLC25A25-AS1, inhibits EMT in CRC cells, possibly through inhibition of ERK and p38 signalling [252]. In addition to SLC25A25-AS1, the lncRNA Nicotinamide Nucleotide Transhydrogenase-antisense RNA1 (NNT-AS1) has also recently been shown to regulate EMT in CRC via the activation of the MAPK/ERK signalling pathway [408]. In prostate cancer, induction of EMT by lncRNA-ATB overexpression was shown to depend partially on the activation of ERK signalling [173]. BANCR has also been found to induce EMT and migration through the MAPK/ERK signalling pathway in both CRC and endometrial cancer [270,271]. MALAT-1 knockdown significantly reduced MAPK/ERK signalling in gallbladder cancer cells [95], and when functioning as a tumour suppressor in glioma MALAT-1 in glioma acts by attenuating ERK/MAPK mediated signalling [409]. Knockdown of lncRNA BC087858, which promotes EMT in NSCLC cells, is associated with decreased MAPK/ERK signalling [232].

In HCC, the lncRNAs CCHE1 and DBH-AS1 promote carcinogenesis via activation of the ERK/MAPK pathway [363,410], but whether they also utilise this signalling pathway to regulate EMT remains to be resolved.

### 4.4. PI3K/Akt Signalling

PI3K/Akt signalling has been shown to be an element in lncRNA mediated regulation of EMT. For instance, downregulation of MALAT-1 inhibits PI3K/Akt signalling in osteosarcoma cells [96], whilst in contrast, in breast cancer cells, MALAT-1 knockdown was found to lead to increased PI3K/Akt signalling and EMT induction [97]. The effects of lncRNA-ATB on EMT in part depend on the activation of PI3K/Akt signalling [173]. Likewise, the induction of H19 and EMT by TGF-β also appears to involve PI3K/Akt signalling [284]. In NSCLC, several lncRNAs appear to utilise PI3K/Akt/mTOR. UCA1 activates Akt/mTOR signalling to promote EMT in EGFR-mutant NSCLC [194], whilst knockdown of lncRNA BC087858 in NSCLC inhibited the activation of the PI3K/Akt and MEK/ERK pathways and epithelial-mesenchymal transition (EMT) via up-regulating ZEB1 and Snail, and restored sensitivity to gefitinib in NSCLC which had acquired resistance to this targeting agent in the absence of T790M mutation of the EGFR [232].

Linc00152 is an lncRNA which has now been shown to bind directly to EGFR causing an activation of PI3K/Akt signalling in gastric cancer [147]. Indeed, this lincRNA has also been shown to also utilize the mTOR signalling pathway in HCC [146]. From the above it appears clear that PI3K/Akt signalling plays important roles in lncRNA mediated regulation of EMT (Table 1).

### 4.5. HIF-1α Signalling

Hypoxia is a central element in solid tumours, and has been associated with both the “Hallmarks of Cancer” [411], and the Warburg Effect [412]. Hypoxia also however affects the non-coding RNA transcriptome [413] including lncRNAs [414]. The hypoxia-inducible factor-1 (HIF1) signalling pathway activated in response to hypoxia is also important to EMT activation [415,416], and recent evidence demonstrates that this signalling pathway is utilised by lncRNAs in the regulation of EMT (Table 1). For instance, UCA1, which promotes EMT in bladder cancer cells [185], was found to be induced in bladder cancer cells under hypoxic conditions by the direct binding of HIF-1α to its promoter [417]. Several other lncRNAs have now been shown to be regulated by hypoxia such as H19 [107] and linc-RoR [136]. Morevover linc-ROR itself directly regulates the expression of HIF-1α, and it is thought to do so by acting as a ceRNA for miR-145 [136]. Another lincRNA, lincRNA-p21 is also directly induced by HIF-1α under hypoxic conditions, but in this instance utilises a positive feedback loop between itself and HIF-1 α to promote glycolysis under hypoxia [256]. linc00152 also utilises HIF-1α to promote EMT in the metastasis of gallbladder cancer [148], by acting as a ceRNA for miR-138 resulting in the upregulation of HIF-1α, and subsequent progression of EMT. LncRNAs can also prevent the activation of HIF-1α, as the lncRNA CPS1-IT1 was found to inhibit HIF-1α activation via an interaction the protein chaperone Hsp90. This interaction may explain the potential role of this lncRNA in the suppression of EMT [262].

### 4.6. p53

In addition to its various other functions as a tumour suppressor, p53 and its truncated variants also play important roles in the suppression of EMT [418,419]. As mentioned in previous sections, several anti-EMT miRNAs are induced by p53 [65,66,67]. In addition, p53 also promotes the degradation of Slug and expression of E-cadherin [420]. Additionally, p53 and Twist interact and reciprocally negatively regulate expression of each other’s target genes [421].

p53 also plays important regulatory roles with respect to lncRNAs. For example, the anti-EMT lncRNAs TUSC7, GAS5 and lincRNA-p21 are all induced by p53 [235,248,257], while pro-EMT H19 is repressed by p53 [107]. Induction of lincRNA-p21 was shown to be directly involved in the repression of hundreds of genes by p53, repressing genes by associating with hnRNP-K [257]. In this regard, lincRNA-p21 has now been shown to direct p53 binding to p53 regulated promoters [258].

In addition, EMT-regulating lncRNAs have also been found to regulate p53 expression. Linc-ROR has been shown to interact with heterogeneous nuclear ribonucleoprotein I (hnRNP 1) to prevent p53 translation, possibly by preventing hnRNP 1 binding to an internal ribosome entry site in the 5′ UTR of p53 mRNA [134]. MALAT-1 also seems to negatively regulate p53 expression, as MALAT-1 knockdown led to increased levels of p53, as well as of p21 in pancreatic cancer cells [275]. Thus, p53 opposes EMT in part through its regulation of lncRNAs, while some lncRNAs may promote EMT in part through their inhibition of p53.

PVT1 overexpression has been found to inhibit expression of p21, a major effector of p53 dependent cell cycle arrest, with downregulation of p21 being involved in the induction of EMT in pancreatic cancer cells [327]. Unexpectedly, however, given PVT1’s described pro-tumourigenic activity, p53 binds to a canonical response element in the *PVT1* locus and induces transcription, especially in response to DNA damage. It should be noted that several miRNAs are also encoded at the *PVT1* locus and that one of these, miR-1204, could promote accumulation of p53 and either apoptosis or cell cycle arrest when ectopically expressed. It thus appears that the *PVT1* locus may exert opposing effects on p53 signalling through different ncRNAs encoded at this locus [422].

### 4.7. Other Pathways

Finally, lncRNAs can regulate EMT using additional pathways. HOTAIR and ZFAS1 have both been found to promote Notch signalling, while, as already mentioned, lincRA-p21 inhibits Notch signalling [255,423,424]. PVT1 activates Hedgehog signalling through indirectly regulating methylation of PTCH1 via miR-152 [328], while lncRNA-HH also activates Hedgehog signalling to promote EMT, possibly via promoting expression of GAS1, an enhancer of Hedgehog signalling [231,358]. Lastly, and as mentioned already, lncTCF7 appears to be involved in IL6-STAT3 signalling induction of EMT, while HOTAIR also likely activates STAT3 signalling via sponging of miR-7 [119,141].

## 5. LncRNAs, EMT and the Cancer Stem Cell Phenotype

Cancer stem cells (CSCs) are a subpopulation of tumour cells, which are capable of self-renewal, have unlimited replicative potential, are resistant to a wide number of therapies and may even, be the cells that ultimately give rise to metastases [425,426]. Since the existence of CSCs was proposed, they have attracted much attention and it has been speculated that the inability of current therapies to effectively kill CSCs explains the frequent relapse of solid tumours following treatment [426,427]. In addition to promoting invasion and metastasis, EMT is also being recognised to be instrumental in the acquisition of the CSC phenotype [428,429]. Given the emerging roles of lncRNAs in EMT during tumourigenesis, it is unsurprising that many of these lncRNAs are also beginning to be associated with regulation of the CSC phenotype (Figure 1, Table 1).

A small set of key transcription factors, including SOX2, Oct4 and Nanog, are known to be pivotal in maintenance of the pluripotent embryonic stem cell state [430,431]. These pluripotency transcription factors are also being recognized to be highly important to the generation of CSCs, and also to function in the activation of EMT during tumourigenesis [432,433,434].Various EMT-regulating lncRNAs have now been found to promote the CSC phenotype through regulation of these pluripotent stem cell transcription factors (Table 1).

MALAT-1 is upregulated in pancreatic cancer CSCs and knockdown of MALAT-1 decreases the pancreatic CSC fraction [92]. MALAT-1 knockdown was found to reduce levels of SOX2, suggesting regulation of SOX2 by MALAT-1 contributes to the CSC phenotype in pancreatic cancer. Interestingly, MALAT-1 acts as a ceRNA for both miR-200c and miR-145, both of which target SOX2, and thus may act as a ceRNA for these miRNAs to increase SOX2 expression, although this is yet to proven [92]. Loss of MALAT-1 in a glioma stem cell line is associated with loss of expression of SOX2 and Nestin, both essential stemness markers [435].

Moreover, chemical induction of aldehyde dehydrogenase 1A1 (ALDH1A1), a key regulator of stemness, is associated with up-regulated expression of stem cell markers, EMT associated genes and the long non-coding RNAs (HOTAIR and MALAT-1) [436].

HULC has also been shown to affect stemness by cooperating with MALAT-1 to promote liver cancer stem cells growth through binding and loading of the promoter for telomere repeat-binding factor 2(TRF2) [183].

Knockdown of H19 was found to downregulate SOX2, Oct4 and Nanog, as well as other cancer stem cell markers in glioblastoma and embryonic carcinoma cell lines [112,113]. Furthermore, lncTCF7 was demonstrated to be highly expressed in HCC and NSCLC stem cells and to be important for their self-renewal, with lncTCF7 knockdown decreasing the CSC fraction and depleting levels of SOX2, Oct4 and Nanog [140,142]. Linc-ROR appears to function as a ceRNA for miR-145 to de-repress the miR-145 targets Oct4, SOX2 and Nanog [132] while overexpression of this lncRNA can further induce EMT in breast cancer cells by acting as a ceRNA for miR-205 and preventing the degradation of ZEB2, generating cells with stem cell-like properties [133]. This lncRNA has now also been shown to regulate lncRNA key stemness transcriptional factors, such as Oct4, SOX2, and Nanog and affect the CSC population in gastric cancer [437].

Linc00617 is another lncRNA upregulated in breast cancer and its overexpression is associated with an increased breast cancer CSC fraction via upregulation of SOX2 [233]. HOTAIR expression has been found to be necessary for the maintenance of the CSC phenotype in colon and breast cancer cell lines [121], and has recently been shown to regulate the breast cancer CSC population by transcriptionally inhibiting miR-34a, resulting in the upregulation of SOX2 [126]. Interestingly, miR-7, which is inhibited by HOTAIR through the suppression of HoxD10, is downregulated in breast cancer CSCs and overexpression of miR-7 could both partially reverse EMT and decrease the size of the CSC population in breast cancer cell lines by suppressing STAT3 signalling [119]. HOTAIR may therefore promote the adoption of a CSC phenotype through inhibition of miR-7 and activation of STAT3 signalling [119].

Activation of various other signalling pathways by EMT-regulating lncRNAs has also been implicated in the generation of CSCs. Overexpression of lncRNA-Hh, an lncRNA activated by Twist, in breast cancer cells increased Hedgehog signalling, a pathway critical to CSC maintenance [231]. LncRNA-Hh overexpression also activated EMT, elevated levels of SOX2 and Oct4 and enhanced mammosphere-formation ability, while silencing of lncRNA-Hh reversed these effects, suggesting the Twist-lncRNA-Hh pathway is a crucial link between EMT and the CSC phenotype [231]. Likewise, lncRNA modulation of β-catenin signalling has also been implicated in CSCs. The Anti-EMT lincRNA-p21 was found to be downregulated in CRC and glioma CSCs relative to non-CSC cancer cells [253,260] and re-introduction of this lncRNA dramatically suppressed the self-renewal and tumourigenicity of CSCs both in vitro [253,260], and in vivo by inhibition of β-catenin signalling [260]. Finally, β-catenin signalling was also shown to be involved with a HULC/CUDR mediated regulation of human embryonic stem cells (ESC) differentiation into hepatocyte-like cells [182]. CUDR itself has been shown to promote liver cancer stem cell growth and liver stem cell malignant transformation both in vitro and in vivo via upregulation of TERT and C-Myc [203]. The mechanisms by which both CUDR and HULC/CUDR complexes evoke their effects appear to be via epigenetic remodelling of critical promoters [182,202,203], suggesting that novel links exist between lncRNs and epigenetic regulation in cancer stem cells.

## 6. LncRNAs, EMT and Drug Resistance

EMT is also known to contribute to the development of resistance to various cancer therapies [438,439,440]. Indeed, it is being realized that EMT, generation of cancer stem cells and the acquisition of drug resistance by tumours are all intricately interconnected processes [441,442]. As such, the emerging role of lncRNAs in both EMT and the CSC phenotype thus points to them also contributing substantially to the development of drug resistance (Figure 1, Table 1). Indeed, in the following sections we discuss how dysregulation of several EMT-associated lncRNAs has been linked to resistance to various anti-cancer drugs, including classic chemotherapeutic drugs as well as targeted therapies.

### 6.1. EMT LncRNAs and Platinum Based Resistance

Numerous EMT lncRNAs have been implicated in cisplatin resistance (Table 1). For example, the expression of HOTAIR was found to be dramatically upregulated in cisplatin-resistant lung adenocarcinoma cells and expression of HOTAIR by patients’ tumours was negatively correlated with response to cisplatin treatment. Knockdown of HOTAIR could restore cisplatin sensitivity in vitro and in vivo in NSCLC [122,443], and has been shown to restore sensitivity to cisplatin (and doxorubicin) in HCC cells [444]. HOTAIR uses diverse mechanisms one of which involves activation of Wnt/β-catenin signalling in ovarian cancer [122]. Another means by which HOTAIR promotes cisplatin resistance is through modulation of miRNA. In one example, modulation of miR-326/SP1 pathway by HOTAIR was shown to reverse chemoresistance of lung adenocarcinoma cells [445], while in the development of cisplatin resistance in gastric cancer HOTAIR also uses a mechanism that activates the PI3K/AKT/MRP1 pathway via inhibition of miR-126 expression [446]. HOTAIR can also affect sensitivity to cisplatin-based therapy by functioning to downregulate the cyclin dependent kinase inhibitor p21 [123]. Moreover, a DNA methylation signature associated with HOTAIR expression was significantly associated with poor survival in carboplatin-treated ovarian cancer patients [125], suggesting that targeting this lncRNA could be an effective strategy in overcoming resistance to platinum based therapies [124,125].

UCA1 is another lncRNA with an established role in platinum-based resistance. In bladder cancer expression of this lncRNA is elevated following cisplatin treatment and knockdown of UCA1 re-sensitizes bladder cancer cells to cisplatin [189]. Upregulation of this lncRNA has also been observed in oesophageal squamous cell carcinoma [447], ovarian cancer [199] and gastric cancer [197]. In a manner similar to that observed for HOTAIR, UCA1 has also been shown to regulate miRNAs (in this instance miR-27b) as part of a multi-drug resistance to various chemotherapies (including cisplatin) in gastric cancer [197]. However, similar to the observations discussed in previous sections with respect to regulation of EMT, tissue context can also be important in lncRNA roles in drug resistance/sensitivity. For instance, in bladder cancer, UCA1 is associated with sensitivity to drug therapy as knockdown of this lncRNA led to decreased chemosensitivity to a cisplatin/gemcitabine combination, which again involved a miRNA, in this instance miR-196a-5p [186].

Overexpression of H19 has been found to confer cisplatin resistance in high-grade serous ovarian cancer through promotion of gluthathione metabolism [108]. Moreover, this correlation between high expression of H19 and poor response to cisplatin has also been observed in lung cancer [448]. The overexpression of HULC also has been shown to inhibit cisplatin mediated responses in HCC [178], while silencing of this lncRNA can increase the sensitivity of gastric cancer cells to cisplatin [180]. In contrast, re-expression of the lncRNA GAS5 has recently been shown to sensitize resistant NSCLC cells to cisplatin [239].

A plethora of lncRNAs has now been identified as playing functional roles in resistance to oxaliplatin. In a study of response to chemo-resistant HCC cells, Yin et al. identified 61 up-regulated and 59 down-regulated lnRNAs (fold change > 2, *p* < 0.05) associated with resistance [449]. Other lncRNAs identified include Linc00152 [151], SNHG5 [450], MALAT1 [451], CRNDE [452] and HULC [181]. Clearly, lncRNAs play important roles in resistance mechanisms to platinum-based drugs and could conceivably become therapeutic targets for re-sensitization strategies.

### 6.2. EMT LncRNAs and Resistance to Other Chemotherapeutic Drugs

Resistance to other chemotherapeutic drugs has also been associated with EMT associated lncRNAs (Table 1). LncRNA-ROR expression was found to impart resistance to paclitaxel and 5-FU in MDA-MB-231 breast cancer cells [453]. H19 has been shown to contribute to doxorubicin resistance in HCC cells via regulation of DNA methylation of the promoter of the efflux pump P-glycoprotein [109]. Additionally, H19 is overexpressed in temozolomide-resistant glioma cell lines and tumours, with knockdown of H19 increasing sensitivity in glioma cell lines [110]. Knockdown of MALAT-1 increased sensitivity to gemcitabine in pancreatic cancer cells [92].

On the other hand, expression of anti-EMT lncRNAs can promote sensitivity to various chemotherapeutic drugs. Knockdown of GAS5 resulted in increased resistance to docetaxel and 5-FU in breast cancer cells [240]. Additionally, GAS5 overexpression could sensitize breast cancer cells to doxorubicin combined with UV treatment [241], and the role of GAS5 in re-sensitizing cells to doxorubicin has since been confirmed in bladder cancer [242]. Induction of GAS5 has been shown to occur in glioma and colorectal cancer cells treated with doxorubicin [454,455]. Overexpression of anti-EMT lncRNA LEIGC was found to increase sensitivity of gastric cancer cells to 5-FU, while LEIGC knockdown decreased sensitivity [251]. Overexpression of SLC25A25-AS1 decreased resistance to 5-FU and doxorubicin in CRC cells while downregulation increased resistance [252].

Several EMT-associated lncRNAs have also been associated with sensitivity/resistance to Adriamycin including UCA1 [197,198], GAS5 [238], and lnc-ROR [456]. UCA1 has also been linked to docetaxel resistance in prostate cancer [200].

### 6.3. EMT lncRNAs and Resistance to Targeted Breast Cancer Therapies

Altered expression of EMT-associated lncRNAs has also been implicated in resistance to targeted breast cancer therapies (Table 1). Tamoxifen has been a mainstay chemotherapy utilized in breast cancer since its approval in the 1980s [457]. The lncRNA UCA1 has now been identified as a critical mediator of tamoxifen resistance in breast cancer by various mechanisms including sponging of miR-18a, a negative regulator of HIF1α [195], mTOR signalling [458], exosomal delivery of the lncRNA from resistant to sensitive cells [201], or via the Wnt/β-Catenin Pathway [190]. Other EMT associated lncRNAs which have been shown to affect tamoxifen sensitivity in breast cancer include MALAT-1 [99], lncRNA-ROR [459], and HOTAIR [460].

High expression of lncRNA-ATB has been demonstrated to be correlated with trastuzumab resistance in breast cancer patients, being the most upregulated lncRNA in tumour tissue from these patients [168]. Interestingly, miR-200, which suppresses EMT and is inhibited by lncRNA-ATB, is downregulated in trastuzumab resistant breast cancer and cells and moreover, re-expression of miR-200c could restore trastuzumab sensitivity to these cells [174]. Therefore, lncRNA-ATB may mediate trastuzumab resistance via inhibition of miR-200 and induction of EMT. Finally, the lncRNA GAS5 has also been linked to trastuzumab resistance in breast cancer [461]. Overall, it is clear from the emerging data that these lncRNAs are becoming important new candidates, which may have theranostic applications in breast cancer management [462].

### 6.4. EMT lncRNAs and Resistance to EGFR Tyrosine Kinase Inhibitors

EMT has been strongly associated with reduced response to EGFR tyrosine kinase inhibitors (EGFR-TKIs) and it has been suggested that EMT mediates resistance via upregulation of the PI3K-Akt pathway and reduced dependence on the MAPK/Erk pathway [463,464]. It is interesting then that lncRNAs UCA1, BC087858 and GAS5 have all been associated with EMT, PI3K-Akt signalling and resistance to EGFR-TKIs (Table 1). UCA1 is upregulated in the tumours of NSCLC patients with acquired resistance to the EGFR-TKI gefitinib, but lacking the T790M mutation, compared to levels prior to gefitinib treatment. This upregulation is significantly associated with shorter progression-free survival in this patient group following gefitinib treatment. Gefitinib resistance may possibly be mediated by activation of AKT/mTOR signalling and EMT by UCA1. siRNA knockdown of UCA1 re-sensitized tumour cells to gefitinib in vitro and in vivo and inhibited EMT and AKT/mTOR pathway signalling activation [194].

LncRNA BC087858 has been shown to be upregulated in NSCLC cell lines with acquired resistance to erlotinib compared to sensitive or intrinsically resistant cell lines and similarly to be upregulated in patients who developed resistance to erlotinib compared to levels prior to EGFR TKI treatment. Moreover, in patients lacking the T790M mutation, but not in patients possessing the mutation, BC087858 overexpression was significantly associated with shorter progression-free survival following gefitinib treatment. Knockdown of BC087858 was found to upregulate E-cadherin while downregulating vimentin, Snail and Zeb1, and to result in reduced PI3k/Akt signalling, suggesting it may mediate EGFR TKI resistance via activation of EMT and the PI3k/Akt pathway [232]. Furthermore, BC087858 knockdown could resensitize NSCLC cells with acquired resistance to gefitinib lacking the T790M mutation [232].

GAS5 was found to be downregulated in EGFR-TKI resistant lung adenocarcinoma cell line A549 compared to sensitive cell lines and GAS5 overexpression could greatly sensitize A549 cells to gefitinib and GAS5 overexpression in A549 xenograft mouse models potentiated gefitinib treatment [237]. Moreover, overexpression of GAS5 decreased Akt signalling [237,465]. Therefore, lncRNA regulation of Akt signalling seems to highly important in determining the sensitivity of NSCLC cells to the EGFR-TKI gefitinib.

## 7. Conclusions

A greater understanding of the molecular mechanisms governing EMT remains an imperative for the development of novel therapies, which can slow or prevent metastasis, the current great unmet need of cancer therapy. To this end, lncRNAs have emerged as integral players in the complex signalling network governing the activation of EMT in tumourigenesis and metastasis (Figure 1, Table 1). These EMT-regulating lncRNAs are active participants in the major signalling pathways governing EMT, including the TGF-β, Wnt/β-catenin, MARK/ERK, PI3K/Akt and HIF pathways. Given the connection between EMT and the generation of cancer stem cells (CSCs) [425,427,429,466] and development of anti-cancer drug resistance [440,441,442], it is unsurprising then that many of the lncRNAs involved in EMT regulation are also being implicated in these two processes as well (Figure 1, Table 1).

Thus far, two major mechanisms have emerged for how these lncRNAs regulate EMT: (1) epigenetically silencing EMT-related genes, particularly E-cadherin, via recruitment of the polycomb repressor complex (PRC2); and (2) post-transcriptionally by acting as competing endogenous RNAs (ceRNAs) for miRNAs that target genes involved in EMT regulation, especially EMT-TFs. Besides these two mechanisms, others have also been identified, including the direct interaction of lncRNAs with proteins involved in EMT [243,250,264] and regulation of mRNA translation [154]. For many of the lncRNAs identified so far, exactly how they regulate EMT is unknown (Table 1). However, given the diverse functions of lncRNAs at all levels of gene regulation, it is very likely that new mechanisms through which lncRNAs regulate EMT will soon be uncovered.

A very intriguing emerging field of research is the role of exosomes, small extracellular vesicles derived from cells which can transfer molecules from one cell to another, in intercellular signalling during cancer progression. The role of exosomal transfer of lncRNAs in the regulation of EMT is only beginning to receive attention. Elevated levels of ZFAS1 were detected in serum exosomes of gastric carcinoma patients, with high exosomal levels associated with lymph node metastasis and advanced TNM stage. Importantly, it was shown that the transfer of exosomes from ZFAS1 high expression to ZFAS1 low expression gastric cancer cells lead to increased expression of ZFAS1 in the low expression cells, a decrease in epithelial markers and an increase in mesenchymal markers [424]. Several other lncRNAs involved in EMT regulation including MALAT-1, HOTAIR, lincRNA-p21, GAS5, TUG1, UCA1 and H19, have recently been discovered in exosomes [201,467,468] and thus the role of exosomal transfer of these lncRNAs in EMT regulation merits further investigation.

It is interesting that many more lncRNAs have been found to promote EMT than inhibit it, with the converse being true for microRNAs. It is not immediately apparent why this should be the case, although it does fit with the idea that antagonization of anti-EMT miRNAs is one of the major mechanisms of EMT promotion by the pro-EMT lncRNAs.

In conclusion, EMT-regulating lncRNAs are emerging as critical regulators of tumour progression, metastasis and drug resistance via governing EMT. Further study will identify additional lncRNAs involved in EMT as well as shed more light on the molecular mechanisms by which they regulate EMT and the signalling pathways in which they participate, greatly enhancing our molecular understanding of the EMT process in tumourigenesis and possibly even establishing EMT lncRNAs as new therapeutic targets in anti-cancer therapy.

## Figures and Tables

**Table 1:** LncRNAs regulating EMT, the molecular mechanisms and signalling pathways through which they act and their influence on drug resistance and cancer stem cells.

Pro-EMT LncRNAs	Molecular Mechanisms of Action in EMT	Signalling Pathways Involved	Drug Sensitivity/Resistance	Cancer Stem Cells
MALAT-1 (NEAT2)	Acts as ceRNA for miR-1 [89] and miR-204 [90] to derepress Slug; also acts as a ceRNA for miR-205 [91] and predicted to bind miR-200c and miR-145 (in silico analysis) [92]; recruits EZH2 and Suz12 to *CDH1* [91,93]	TGF-β [93], Wnt [91,94], MAPK [95], PI3K/Akt [96,97], p53 [98]	Tamoxifen [99], Gemcitabine [100]	Pancreatic Cancer [92]
H19	Acts as a ceRNA for let-7 [101], miR-138 [102], miR-200 family members miR-141 [103] and miR-200a [102] and miR-630 [104] and recruits EZH2 to CDH1 [105] and NKD1 [105,106]	TGF-β [106], Wnt [105], p53 [107]	Cisplatin [108], Doxorubicin [109], Temozolomide [110]	Glioblastoma [111,112], Embryonal Carcinoma [113] Liver Cancer [114]
HOTAIR	Recruits PRC2 to *CDH1* [115], miR-34 [116], miR-568 [117] and *WIF1* [118]; indirectly inhibits miR-7 through suppression of HoxD10 [119], and as part of a SNAIL/EZH2 tripartite complex to EMT genes [120]	TGF-β [121], Wnt [118,122]	Cisplatin [122,123,124] Carboplatin [125]	Breast Cancer [119,121,126], Colon Cancer [121] Colorectal Cancer [127] Liver Cancer [128,129] Lung Cancer [130] Ovarian Cancer [131]
Linc-ROR	Acts as a ceRNA for miR-145 [132] and miR-205 [133]; Possibly through interaction with hnRNP 1 to prevent p53 translation [134] Upregulates ZEB1 [135], and prevents degradation of ZEB2 [133]	HIF [136], p53 [134]	5-FU [137], Paclitaxel [137] Gemcitabine [137,138]	Breast Cancer [133] various cancers [139]
lncTCF7	Possibly through recruitment of Swi/SNF to the TCF7 promoter, triggering TCF7 expression and activating Wnt signalling [140]	Wnt [140], IL-6/STAT3 [141]	-	HCC [140] NSCLC [142]
Linc00152	Acts as a ceRNA for miR-139-5p [143] Binds to EZH2 [144,145]	mTOR [146] EGFR [147] HIF-1α [148] PI3K/Akt [149]	Cisplatin [150] Oxaliplatin [151]	human-induced pluripotent stem cells (hiPSCs) [150]
ZEB1-AS1	Upregulates ZEB1 by acting as ceRNA for miR-200 species [152], or by recruiting histone acetyltransferases to the ZEB1 promoter [153]	-	-	-
ZEB2-AS1	Binds to IRES in ZEB2 mRNA to increase ZEB2 translation [154]	TGF-β [155]	-	-
EGFR-AS1	Unknown	GHR modulates EGFR by regulating EGFRAS1 expression [156]	-	-
HNF1A-AS1	Interacts with DNMT1 to possibly silence *CDH1* [157,158,159] Upstream regulator of H19 [160] Acts as a ceRNA for hsa-miR-30b-5p [161] Interacts with EZH2 [162]	Wnt/β-catenin [163]	-	-
CBR3-AS1 (PlncRNA-1)	Unknown	TGF-β [164] AR [165] HER2 [166]	-	-
LncRNA-ATB	Acts as ceRNA for miR-200 family [167,168,169,170] Acts as a ceRNA for mir-141-3p [171] Suppresses E-Cadherin expression [172]	TGF-β [169], PI3K/Akt [173]	Trastuzumab [168,174]	-
HULC	Acts as ceRNA for miR-200a [175,176] and miR-372 [177] Binds to YB-1 promoting its release cyclin D1, cyclin E1, and matrix metalloproteinase 3 [178] Binds to and stabilizes Sirt1 Interacts with EZH2 [179]	PI3K/Akt [176] ERK [178]	Cisplatin [180] Oxaliplatin, 5-FU and THP1 [181]	Liver Cancer [182,183]
UCA1 (aka CUDR)	Acts as ceRNA for miR-16 [184], miR-145 [185], miR-196a-5p [186], miR-216b [187], and miR-485-5p [188]	Wnt [189,190,191,192,193] MAPK [187], Akt/mTOR [194], HIF-1α [185,195] TGF-β [196]	Adriamycin [197,198] Cisplatin [186,189,197,199] Docetaxol [200] Gefitinib [194] Imatinib [184] Multi-drug resistance [197] Tamoxifen [195,201]	[182,202,203]
TUG1	Acts as ceRNA for miR-145 [204], and mir-300 [205]	TGF-β [205]	platinum-based chemotherapy combined with 5-fluorouracil (FU) or paclitaxel [206]	Glioma [207]
NEAT1	Acts as ceRNA for miR-204 and miR-101 [208,209]	-	Cisplatin [210] multi-drug resistance [211]	Glioma [212,213] Breast Cancer [214]
lncRNA-EBIC	Recruits PRC2 to *CDH1* [215]	-	-	-
CCAT1 (aka CARLo-5)	Interacts with miR-490 [216] Acts as a ceRNA for let-7 [217] Acts as a ceRNA for miR-218-5p [218] Acts as a ceRNA for miR-155 [219]	c-MYC [220,221,222]	Predicts sensitivity to BET inhibitors in colorectal cancer [223]	-
CCAT2	Interacts with EZH2 [224]	CCAT2 has been shown to regulate cancer cell metabolism [225] Wnt [226] TGFβ [227]	Genetic polymorphisms in CCAT2 have been linked to cisplatin resistance [228]	-
lncRNA-HIT	Stabilization of ZEB1 protein [229]	TGF-β [230]	-	-
lncRNA-HH	Directly targets GAS1 [231]	Hedgehog [231]	-	Breast Cancer [231]
BC087858	Unknown	MAPK [232], PI3K/Akt [232]	Gefitinib [232]	-
Linc00617	Possibly through recruiting hnRNP-K to *SOX2* promoter [233]	-	-	Breast Cancer [233]
ZFAS1	**-**	Notch [234]	**-**	**-**
**Anti-EMT LncRNAs**	**Molecular Mechanisms of Action**	**Signalling Pathways Regulated**	**Drug Resistance**	**Cancer Stem Cells**
GAS5	Unknown	P53 [235,236] Inhibits IGF1R signalling [237] BRCA1 [236]	Adriamycin [238] Cisplatin [239], Docetaxel [240], Doxorubicin [241,242], 5-FU [240], Gefitinib [237]	-
GAS5-AS1	Unknown	-	-	-
LncRNA-Dreh	Interacts with vimentin protein and represses vimentin expression [243]	-	-	-
TUSC7	Acts as ceRNA for miR-10a [244] Acts as a ceRNA for miR-211-3p [245] Binds to miR-23b [246] Directly regulates miR-23b [247]	integrin β1 pathway signalling [244] p53 [247,248]	5-FU [249], Cisplatin [249]	-
AOC4P	Promotes ubiquitination and degradation of vimentin protein [250]	-	-	-
LEIGC	Unknown	-	5-FU [251]	-
SLC25A25-AS1	Unknown	MAPK [252]	5-FU [252], Doxorubicin [252]	-
LincRNA-p21	Unknown	β-catenin [253,254], Notch [255], HIF [256], p53 [257,258]	Methotrexate [259]	CRC [260], Glioma [253] preiPSC to iPSC conversion [261]
CPS1-IT1	Possibly through interaction with Hsp90 and inhibition of its activation of HIF-1α HIF-1α [262]	HIF-1α [262]	-	-
Linc00261	Binds to Slug protein and promotes its degradation [263]	-	-	-
**LncRNAs with Variable Roles in EMT**	**Molecular Mechanisms of Action**	**Signalling Pathways Regulated**	**Drug Resistance**	**Cancer Stem Cells**
SPRY4-IT1	Promotes EMT by interacting with Snail and regulating its stability [264] Acts as a ceRNA for miR-101-3p, resulting in up-regulation of EZH2 [265] Interacts with EZH2 to epigenetically repress *CDH1* expression [266]	TGF-β [267]	-	-
Linc01133	Inhibits EMT by interacting with and inhibiting SRSF6 [268]. Promotes EMT by recruiting EZH2 to *CDH1* [269]	-	-	-
BANCR	Unknown	MAPK [270,271] ERK [272] NFκB [273]	-	-
